# A spatially explicit analysis of chronic diseases in small areas: a case study of diabetes in Santiago, Chile

**DOI:** 10.1186/s12942-020-00217-1

**Published:** 2020-06-23

**Authors:** Ricardo Crespo, Claudio Alvarez, Ignacio Hernandez, Christian García

**Affiliations:** 1 Departamento de Ingeniería Geográfica, Universidad de Santiago de Chile, Avenida Libertador Bernardo O’Higgins, 3363 Estación Central, Santiago, Chile; 2 Facultad de Ciencias Médicas, Universidad de Santiago de Chile, Santiago, Chile

**Keywords:** Chronic diseases, Diabetes, Spatial microsimulation, Self-organizing maps

## Abstract

**Background:**

There is a strong spatial correlation between demographics and chronic diseases in urban areas. Thus, most of the public policies aimed at improving prevention plans and optimizing the allocation of resources in health networks should be designed specifically for the socioeconomic reality of the population. One way to tackle this challenge is by exploring within a small geographical area the spatial patterns that link the sociodemographic attributes that characterize a community, its risk of suffering chronic diseases, and the accessibility of health treatment. Due to the inherent complexity of cities, soft clustering methods are recommended to find fuzzy spatial patterns. Our main motivation is to provide health planners with valuable spatial information to support decision-making. For the case study, we chose to investigate diabetes in Santiago, Chile.

**Methods:**

To deal with spatiality, we combine two statistical techniques: spatial microsimulation and a self-organizing map (SOM). Spatial microsimulation allows spatial disaggregation of health indicators data to a small area level. In turn, SOM, unlike classical clustering methods, incorporates a learning component through neural networks, which makes it more appropriate to model complex adaptive systems, such as cities. Thus, while spatial microsimulation generates the data for the analysis, the SOM method finds the relevant socio-economic clusters. We selected age, sex, income, prevalence of diabetes, distance to public health services, and type of health insurance as input variables. We used public surveys as input data.

**Results:**

We found four significant spatial clusters representing 75 percent of the whole population in Santiago. Two clusters correspond to people with low educational levels, low income, high accessibility to public health services, and a high prevalence of diabetes. However, one presents a significantly higher level of diabetes than the other. The second pair of clusters is made up of people with high educational levels, high income, and low prevalence of diabetes. What differentiates both clusters is accessibility to health centers. The average distance to the health centers of one group almost doubles that of the other.

**Conclusions:**

In this study, we combined two statistical techniques: spatial microsimulation and selforganising maps to explore the relationship between diabetes and socio-demographics in Santiago, Chile. The results have allowed us to corroborate the importance of the spatial factor in the analysis of chronic diseases as a way of suggesting differentiated solutions to spatially explicit problems. SOM turned out to be a good choice to deal with fuzzy health and socioeconomic data. The method explored and uncovered valuable spatial patterns for health decision-making. In turn, spatial microsimulation.

## Introduction

The increase of chronic diseases in recent decades is a global concern. According to the World Health Organization (WHO), in 2001, chronic diseases caused the death of about 30 million people, equivalent to 60% of total deaths [1]. In 2016, in a new report, the WHO indicates that chronic conditions are associated with the deaths of 40.5 million people worldwide [2], equivalent to 71% of total deaths. This increasing trend is particularly severe in developing countries [3]. Chronic diseases affect quality of life and have a substantial economic impact on public and private health systems. Naturally, pressure is generated on governments concerning the public policies required to prevent and control chronic diseases. Most prevention plans focus on promoting lifestyle changes related to alcohol consumption, tobacco use, nutrition, and exercise, among others. Access to treatment is usually associated with the capacity and spatial distribution of health network providers. A health network is here understood as a group of primary, secondary and tertiary healthcare establishments in a city.

In this context, public policies aimed at improving prevention plans and optimizing the allocation of resources in health networks should be designed specifically for a population’s social and economic realities using a multi-dimensional approach. One way to tackle this challenge is by exploring the link between the sociodemographic attributes that characterize a community, its risk of suffering chronic diseases, health insurance coverage, and treatment accessibility [4–6]. For this reason, and to support decision making of health planners, particularly in large cities, it is necessary to incorporate the spatial domain to study this link [7, 8]. Exploring the spatial heterogeneity of socioeconomic and health indicators is a valuable tool to design and improve health networks [9, 10].

In large and populated cities, spatial heterogeneity is best explored over a small area (i.e. the neighbourhood level). However, health indicators are rarely available for small areas; for this reason, we suggest the use of spatial microsimulation, a powerful statistical technique used primarily to disaggregate sociodemographic data at different geographical scales. Microsimulation (spatial and non-spatial) has been present in the area of health since the 1970s in various studies on topics such as fertility, private health systems and cancer [11–14]. Most of these studies use census data and specific surveys to generate synthetic data at the neighbourhood level. New research around spatial microsimulation in public health has been applied to study the effects of population ageing and the costs associated with public policies [15], including the need to improve health services for diseases such as diabetes [16] and dementia in the elderly [17].

Due to the inherent complexity of urban systems and to approach the problem from a multidimensional perspective, we propose using a spatial clustering methodology to find spatial sociodemographic patterns that link the population with the prevalence of the most frequent chronic diseases. Specifically, we suggest the use of a clustering method called self-organizing maps (SOM) introduced by Kohonen [18] to determine high-risk populations and their geographical location. SOM, unlike classical clustering methods, incorporates a learning component through neural networks, which makes it more appropriate to model complex adaptive systems (i.e. constantly changing systems) such as cities. For this reason, the SOM method has gained popularity in population studies of large cities where high sociodemographic heterogeneity is present. SOM has previously been used in demography and in public health studies [19–22].

In summary, the main objective of this study is to propose a statistical clustering methodology to support decision-making around the allocation of resources, prevention campaigns and the spatial location of primary public health establishments in the fight against chronic diseases. This type of decision-making process involves the use of different kinds of information, either quantitative or qualitative. In this study, we attempt to contribute to this process through spatially explicit quantitative information. We also attempt to contribute to the literature by creating this methodology in a way that is applicable and adaptable to the reality of other cities. To the best of our knowledge, there are no studies combining spatial microsimulation and SOM to explore patterns of chronic diseases in a spatially explicit manner.

## Methods

### Study area

For the case study, we chose the city of Santiago, Chile. Santiago is the capital and largest city in Chile. The city lies in the country’s central valley and has an approximate population of seven million inhabitants (35% of Chile’s total population). Given its stable economic growth since the 1990s, Chile became an OECD country in 2010. Despite being classified as a high-income country by the World Bank, Chile is ranked the highest in inequality among OECD countries, with a Gini index of 0.47 [23]. Chile is classified as a country with an efficient and well-organized health system, but challenges like the increase of chronic diseases and the ageing population could have a severe impact on the health system and the country’s economy [24]. Chile also has the sixth highest adult diabetes prevalence among OECD countries, as about 10% of Chilean adults are diabetics. The Ministry of Health of Chile, through its 2016–2017 National Health Survey, reported that 12.3% of Chileans suffer from diabetes (about 1.8 million people), and the prevalence increases to 30% in the elderly [25].

Chile has high rates of tobacco and alcohol consumption compared to other OECD countries. Likewise, the obesity rate is 34.4% in adults and 44.5% in children. Chronic diseases have been the leading cause of death in Chile in the last decades [26]. The rise of chronic diseases in the elderly population in the last decade also suggests that there will be an increase in the demand for medical care in the coming decades.

### Data

To characterize the population and as input for the microsimulation, we used data from the 2017 census at the smallest scale available: census zones. These areas usually comprise between 1000 and 5000 people residing in a group of neighbouring blocks. The city of Santiago consists of 34 municipalities, with an average population of 176,000 inhabitants each, and 1643 census zones, with a mean population of 3600 inhabitants. To obtain health data, we used the Chilean Socioeconomic Survey of 2017 (CASEN). The CASEN survey is composed of seven characterization modules for individuals and families: resident registry, education, work, income, health, identities and housing characteristics. Due to the inherent complexity of cities with high socioeconomic heterogeneity, CASEN used a probabilistic, stratified, multistage and conglomerate sample to achieve a good representation of the population according to the socioeconomic diversity in each municipality based on the 2017 census cartographic mapping for the sampling method. Detailed information on the sample design can be found in the official document of the Ministry of Social Development, the public agency in charge of carrying out the survey [27].

Given that diabetes is a rising burden in Chile and because of an adequate response rate to the survey, we selected diabetes in adults as the chronic disease in our study. Chile included diabetes as one of the diseases that qualify for universal access, expenditure protection and a guarantee of access to diagnosis and treatment. Coverage of diabetes and another 32 high-burden diseases was established by law in the health reform of 2004 and applied to public and private insurance [28]. To measure diabetes, we used the CASEN question that requested auto-reporting of diabetes treatment in the last 12 months, which is available at the municipality geographical level. The sample size of the CASEN is 35,228, of which 4.1% reported diabetes.

It is worth pointing out that the CASEN survey does not distinguish between diabetes types I and II in the corresponding answer. However, the fact that about 90 to 95 of people with diabetes correspond to Type II diabetes [29] provides us with the confidence to address the problem in a statistically meaningful manner.

### Spatial microsimulation: Methodology to disaggregate prevalence of diabetes at census zone level

To disaggregate the prevalence of diabetes from the municipality to the census zone level, we used spatial microsimulation. This technique generates a synthetic population by combining census data available at the smaller area scale with data from socioeconomic surveys available at the larger geographical level [30]. The synthetic population can be seen as an enriched version of the census data containing additional socioeconomic attributes, which are typically variables associated with income or health indicators [31, 32]. We used years of education, age and gender as the link variables between the census and the CASEN survey. Spatial microsimulation was performed using an iterative proportional fitting algorithm (IPF) [33] by which the health attributes of each simulated individual were calculated using contingency tables from both data sets. The spatial microsimulation method generates the input data to be used ultimately in the SOM clustering process.

### Self-organizing maps

SOM is an unsupervised neural network method that operates by clustering multidimensional input data and reducing them to a two-dimensional representation. Clusters are formed based on similarities and patterns of a series of attributes from the input data. This method operates similarly to the traditional *k*-*means* clustering method in that it looks for similarities by calculating the Euclidean distance between the input data attributes. SOM’s unsupervised neural network algorithm achieves better classification in processes where the high multidimensionality of the attributes makes it difficult to classify and distinguish between one cluster and another. This type of behaviour is frequently observed in demographic and health phenomena, where people with similar sociodemographic characteristics have different tendencies to suffer from chronic diseases or people with different sociodemographic characteristics may exhibit similar trends.

**Fig. 1:**
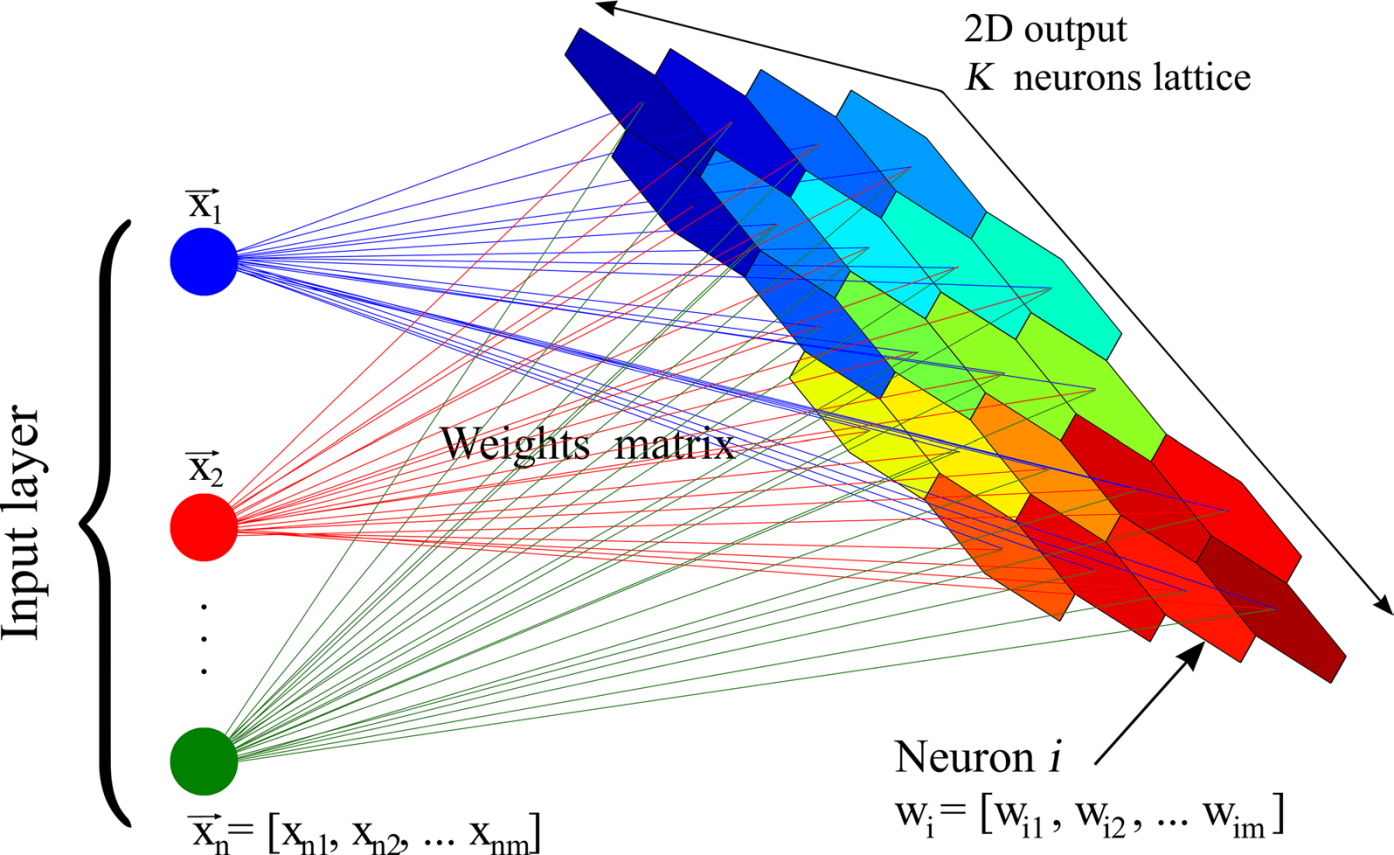
SOM map structure (with permission of authors [34])

Figure 1 shows a concise representation of how the SOM operates obtained from [34]. The input layer includes *n* vectors of input data, each one containing a set of *m* attributes. The output layer is depicted by the coloured grid comprising *K* neurons, each represented by a multidimensional weight vector of length *m*.

The purpose of the algorithm is to group together closely across the 2D grid representation those neurons with a similar combination of input attributes. To quantify the level of similarity of the neurons, the algorithm calculates for each vector of input data $$ \overset{\lower0.5em\hbox{$\smash{\scriptscriptstyle\rightharpoonup}$}} {x}_{n} $$ the Euclidean distance $$ d(X,W) $$ between its standardized attributes and each as follows:

1 $$ d(X,W) = \left\| {X - W_{i} } \right\| = \sqrt {\sum\limits_{j = 1}^{m} {(x_{j} - w_{ij} )^{2} } } $$

where *i: 1,…,K*.

Each input vector is assigned the neuron associated with the smallest Euclidian distance (the winning neuron). Finally, clusters are formed by grouping together neurons with similar values; that is, similar colours in Fig. 1. This secondary clustering is usually done via hierarchical clustering techniques. A key point in the SOM process is the updating of the initial values of the weights of each neuron. As these values are first unknown, they must be initialized, usually by assigning small random values. To find meaningful clusters, these values must thus be updated at each iteration. Basically, the weight vectors of the winner and its neighbouring units in the output space are adjusted to become more representative of the features that characterize the input space.

This clustering method is applied to each of the 1643 census zones (index *m,* as indicated above). Each census zone must thus be previously characterized on the basis of a series of attributes that correspond to the input variables for the SOM algorithm. The simulated population of each census zone is characterized according to the following seven attributes:

1) Education: Percentage of people with professional studies.

2) Low income: Percentage of people with low income, where low income is defined as below US$360, corresponding to the median of income distribution in the study area.

3) Sex: Percentage of people who are male.

4) Age: Percentage of people in the age ranges of 30–45 years and 46–60 years. These age ranges leave children and older adults out of the analysis. Diabetes in children differs from the adult. Children mostly tend to develop diabetes mellitus (DM) type I, caused by a reduced insulin production, due to genetic and immune causes. DM II, which more often appears in adults and the elderly, is associated with resistance to insulin in the body peripheral cells related to lifestyle and habits. Accordingly, including children in the analysis could lead us to draw erroneous conclusions. We have also excluded older adults because they are probably not receiving income, which could also lead to erroneous conclusions. Finally, we have concentrated on adults over the age of 29, an age by which they would have finished their professional studies if they had taken them.

5) Accessibility: Euclidean distance from the centroid of the census zone to the nearest primary public health service within the municipality where the census zone is located.

6) Public health system: Percentage of people within the public health system.

7) Diabetes: Percentage of people who reported has been under treatment for diabetes in the last 12 months.

## Results

### Spatial microsimulation: Generating the input data at census zone level

**Fig. 2:**
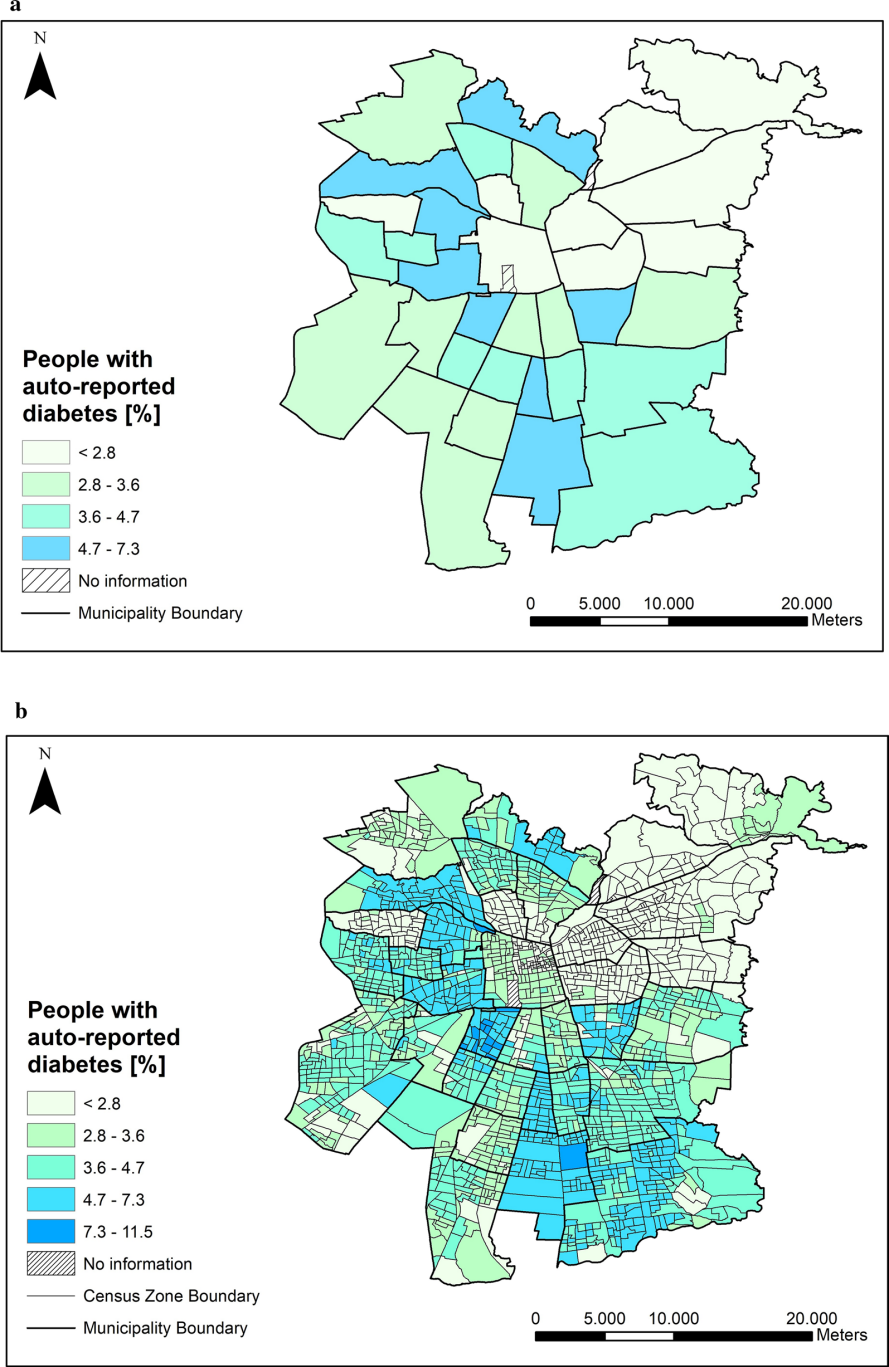
**a** Spatial distribution of diabetes auto report at municipality level. **b** Spatial distribution of diabetes auto report at census zone level

**Fig. 3:**
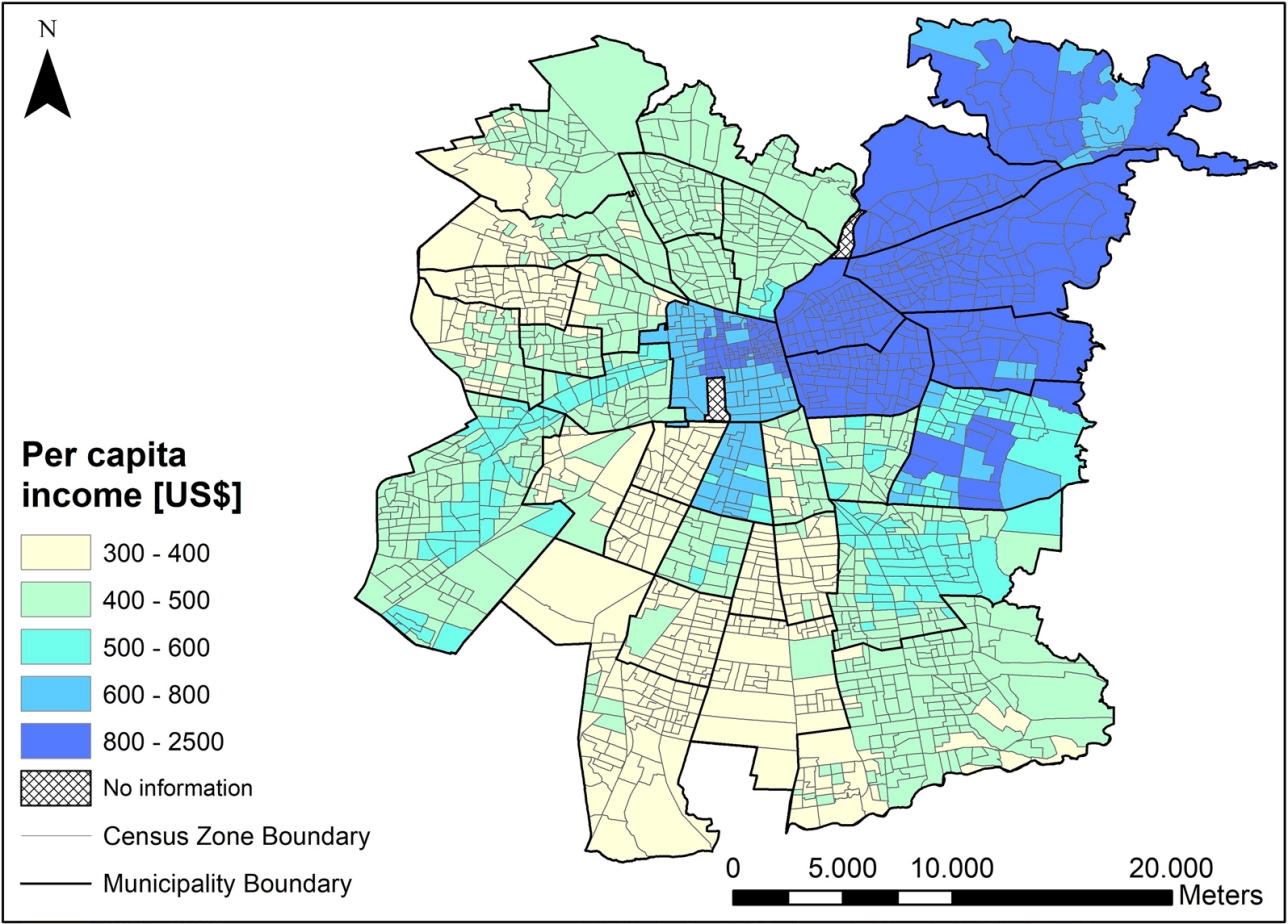
Spatial distribution of per capita income (with permission of authors [30])

As a first exploratory analysis, Fig. 2a, b below show at municipality and census zone level respectively, the spatial distribution of the percentage of people with auto-reported diabetes as obtained from the spatial microsimulation. Both figures include two zones without census information (corresponding to parks). As a reference for a preliminary socio-economical exploratory analysis of the city, Fig. 3 shows the spatial distribution of the average per capita income at the census level obtained from [30].

Figure 2a suggests a city with significant spatial variability in people with auto-reported diabetes, with values ranging between less than 2.8% up to 7.3%. A pattern of low diabetes prevalence can be observed from the city centre towards the northeast, while the rest of the city shows a mix of patterns. In turn, Fig. 2b, also reveals a significant spatial variability at the smaller spatial level. Although a few municipalities (about 10 out of 34) exhibited low or even nil spatial variability, the rest showed such variability that, within some municipalities, diabetes levels even doubled(or more) from one neighbourhood to another.

Concerning the goodness of fit of the spatial microsimulation, we used the standardized absolute error (SAE) as recommended by [35]. This method first requires the calculation of the total absolute error (TAE) as, $TAE = \sum\limits_{{}} {\left| {y - \hat{y}} \right|}$ where $y$ and $\hat{y}$ are the observed and the simulated values, respectively. In our case, these values equal one if the person reported diabetes and zero otherwise. Next, the SAE is obtained by dividing the TAE by the number of observations. In our case, the observed values come from the CASEN 2017 survey, as it contains the information for diabetes prevalence. Concerning the error threshold for rare diseases (e.g. diabetes), the model needs to be accurate at a level less than 10%, that is, an SAE < 0.1 [36]. In our case, we obtained an SAE of 0.067.

Figure 3 shows the significant spatial pattern links in the area of the city with the highest income per capita with a smaller number of people who responded that they had been under treatment for diabetes in the previous year. This might suggest that people with higher incomes have better access to treatment, and it may also indicate that the same people have fewer chances to suffer from diabetes. However, the heterogeneous spatial variability of the number of people with diabetes in the census zones in Fig. 2b differs greatly from the more homogeneous spatial patterns of income in Fig. 3. These patterns support the multifactorial nature of diabetes. In other words, although the per capita income may have an important correlation with the development of diabetes, it does not by itself explain the spatial heterogeneity seen in Fig. 2b. It is therefore necessary to include other sociodemographic variables in the clustering.

### SOM: Clustering of adults with diabetes in Santiago, Chile

To proceed with the SOM method, we used the *Kohonen* package for the R statistical software and followed the guidelines of [37]. Prior to running the algorithm, the number of neurons (*K*) of the grid arrangement has to be determined and this will also determine the number of final clusters as depicted in Fig. 1 (groups of neurons with similar colour tonality). According to Kohonen [38] it is not possible to guess or estimate the exact number of neurons in the array beforehand; this must be determined by the trial-and-error method, after seeing the quality of the first guess. One thus has to find a proper combination of the number of neurons and the number of final clusters to obtain a minimum of meaningful clusters for the study. As a statistical technique, some clusters from the SOM method may appear as a result of a purely mathematical elaboration, without having a straightforward and clear interpretation from a socioeconomic and health viewpoint. This trial-and-error method must be undertaken with care to achieve representative patterns. On the other hand, if the number of neurons is too small, some pattern characteristics might not be represented, while if it is too big, adjacent patterns will be too similar and visualization is unwieldy [39].

In our case, after various trial-and-error iterations, we selected a 10 × 10 grid containing 100 neurons and 12 clusters. To obtain these clusters, the *complete linkage* method for hierarchical clustering was used. Although there are various types of hierarchical clustering methods, we selected *complete linkage* as it produces more compact clusters [40], and this may facilitate the understanding and visualization of the clusters formed and displayed on the 2D grid with 100 neurons. More detailed information on hierarchical clustering methods can be found in [41]. Even using the complete linkage method, however, some neurons associated with a cluster may not ultimately be located in an adjacent position to the cluster. This is usually caused by the random initial values assigned to each neuron. In fact, running the algorithm with new random values is likely to produce a new arrangement of neurons and clusters on the grid. Even so, the final result should remain the same: that is, each cluster will contain the same type and number of neurons.

To ease the final geographical visualization of the results to be displayed on the study area map, we selected only 4 out of the 12 clusters. The use of the 12 clusters may overload the map with colours and make the analysis more difficult. The main criterion for selecting these 4 clusters is the socioeconomic representativeness of our study population, and the 4 selected clusters accounted for nearly 75% of the whole study area population and comprise a wide range of population income and educational levels. In general terms, the 4 selected clusters can be described as follows:

High, medium and low are descriptive labels derived from dividing the range of each variable into three intervals of the same length. A more detailed description of all attributes for each selected cluster, including the median and interquartile range, can be found in Appendix I.

**Fig. 4:**
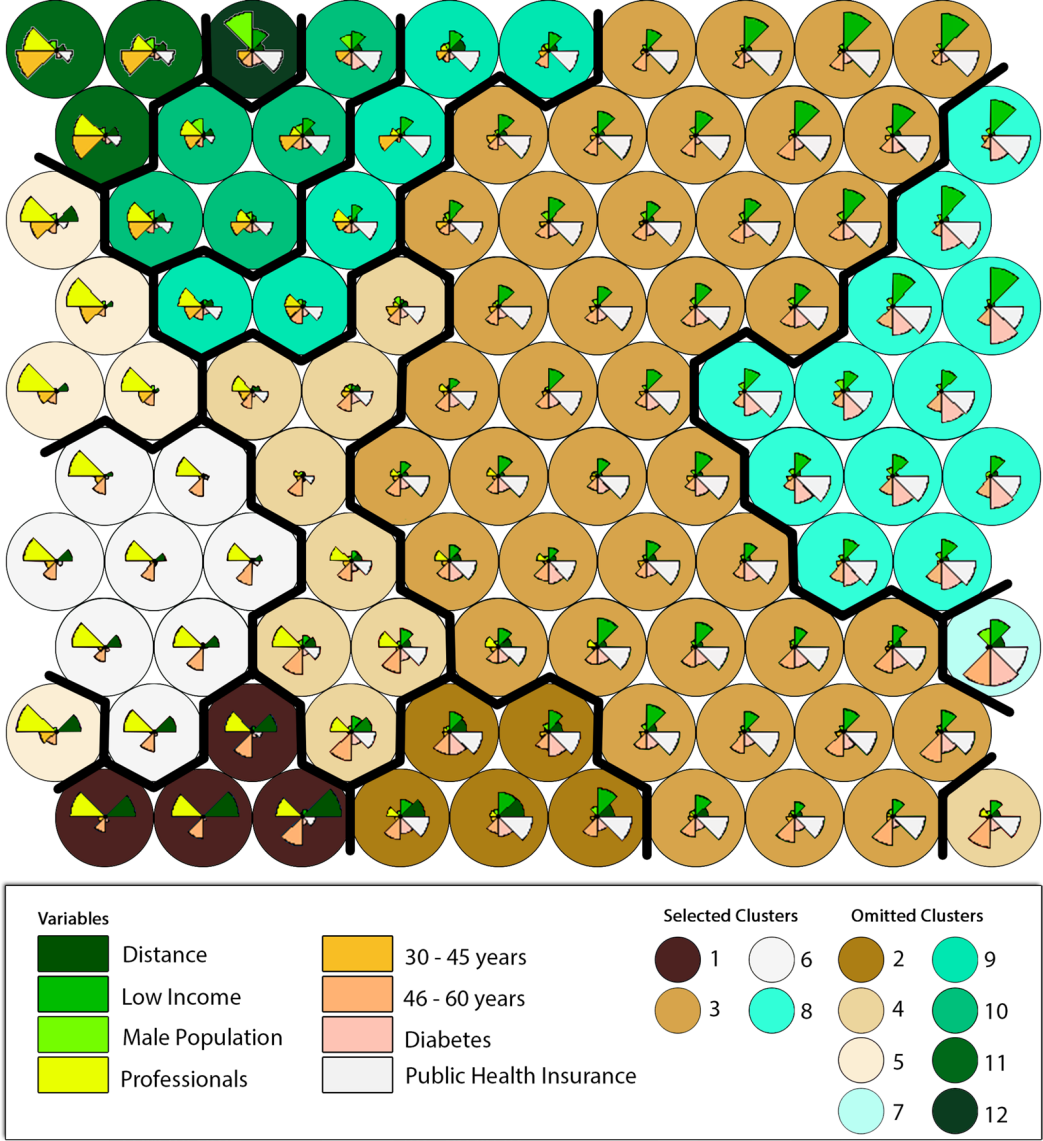
2D grid output of the SOM method]

Figure 4 shows the 12 clusters (selected and omitted) on the 2D grid output of the SOM method applied to the case study. The algorithm converged after 200 iterations. For simplification, the algorithm assigned one simple colour to each cluster. It is worth highlighting that each neuron contains information from a set of census zones, which are not necessarily neighbouring zones geographically.

The figure includes a pie chart inside each neuron summarizing the average values of the input attributes over the grid. In this case *Distance* is associated with the variable *Accessibility*, while *Professionals* with the variable *Education* as described above. As can be observed, neurons of each cluster tend to have a similar distribution of the input values. However, this visualization analysis must be done with care since neurons are ultimate grouped together based on similar mix of attributes calculated the Euclidian distance as indicated in Eq. 1. Accordingly, a cluster might be made up of neurons with a noticeable different combination of input attributes. This is the case of cluster 9 in Fig. 4, where the percent of professional is highly significant for some neurons, while almost nonexistence for some others. A disaggregated and coloured visualization version of the pie chart for each one of the input attributes is known as the plane heat map. These are shown in Appendix II.

**Fig. 5:**
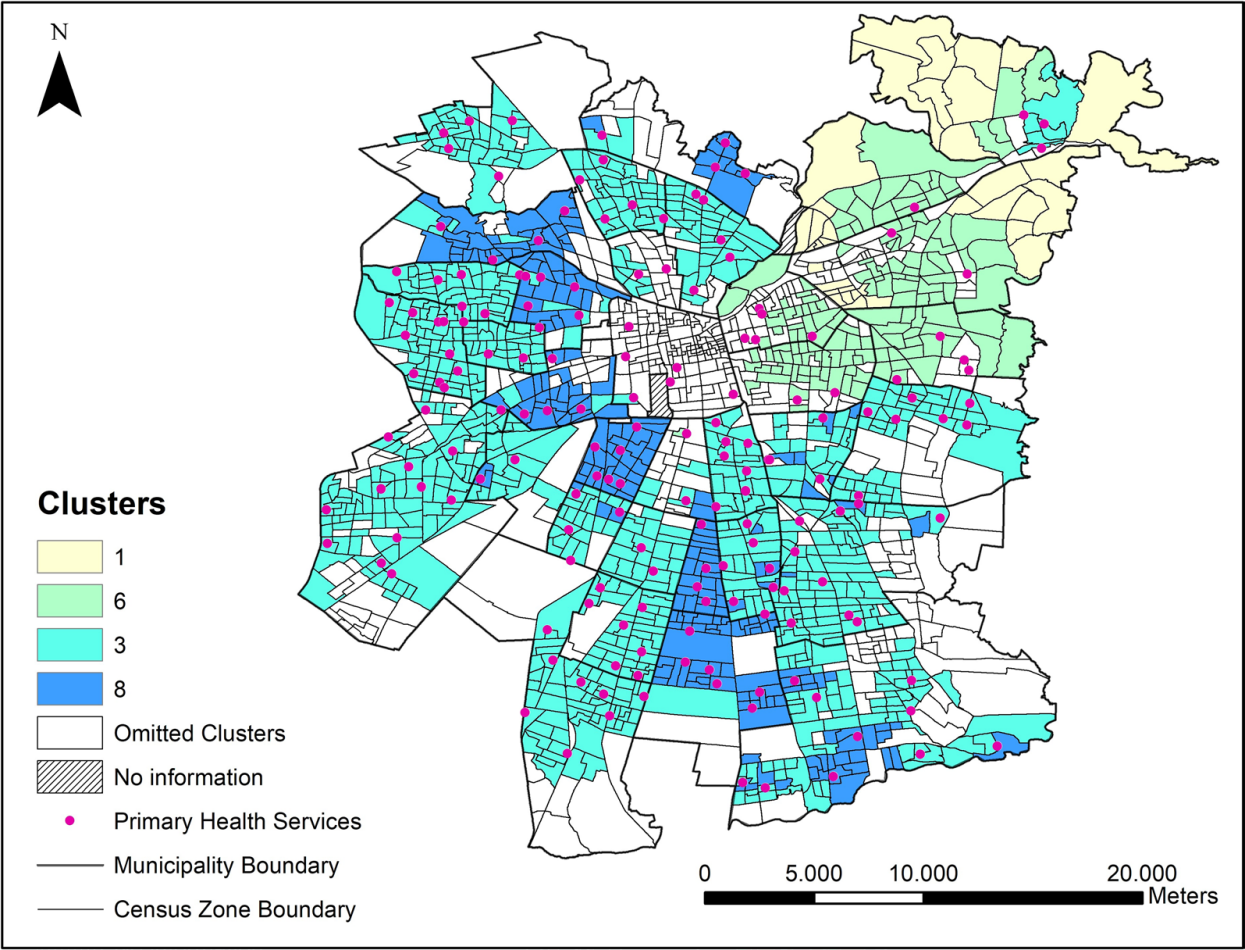
Geographical representation of the SOM results]

The geographical representation of the SOM results is shown in Fig. 5, along with the unexamined areas that correspond to the clusters omitted for this analysis, and the location of primary public health services (as a reference for further analysis). Figure 5 shows the spatial distribution of the clusters detailed in Tables 1 and 2 (see Appendix I).

**Table 1:** Description of selected clusters

Cluster number	Description
1	Low diabetes, high class, high education, low accessibility
6	Low diabetes, high class, high education, medium accessibility
3	Medium diabetes, low class, low education, high accessibility
8	High diabetes, low class, low education, high accessibility

**Table 2:** Description of selected clusters

Cluster number	Diabetes[%][IQR]	Distance[m] [IQR]	Low income[%] [IQR]	Male population[%] [IQR]	Professionals[%] [IQR]	30–45 years[%] [IQR]	46–60 years[%] [IQR]		
**1**	2.21 [2.04–2.43]	3443 [3942–3067]	3.94 [3.62–7.56]	45.30 [44.50–46.39]	44.96 [40.60–47.61]	28.28 [24.73–30.87]	26.08 [24.51–29.72]	10.87 [8.58–23.41]	148,918
**6**	1.80 [1.43–2.17]	1454 [974–1937]	4.34 [2.18–5.44]	45.38 [44.46- 46.33]	43.91 [40.46–46.96]	30.20 [26.62–33.85]	25.65 [24.81–33.85]	15.65 [11.70–23.50]	487,850
**3**	3.88 [3.43–4.34]	657 [410–943]	30.03 [26.01–34.06]	48.51 [47.70–49.27]	7.31 [4.78–11.94]	30.26 [28.56–32.20]	26.13 [24.71–27.64]	81.97 [75.39–86.85]	3,052,920
**8**	5.81 [5.40–6.37]	578 [340–869]	31.72 [28.56–46.39]	49.05 [48.23–49.74]	4.84 [3.69–7.76]	29.27 [27.88–31.32]	25.96 [24.19–26.98]	84.81 [79.22–91.31]	861,270

In general terms, Fig. 5 indicates that clusters 1 and 6 include people with the highest income of the city, the best education level, and the lowest level of diabetes. These clusters are located in the north-eastern part of the city. Clusters 1 and 6 differ from each other in that people from cluster 1 are located farther away from public services than people from cluster 6. In addition, about 15% of people from cluster 6 have public health insurance compare to the 10% of people from cluster 1. Compared to clusters 1 and 6, clusters 3 and 8 have people with lower income, lower education, higher accessibility to public health services, and higher levels of diabetes.

Cluster 3 is geographically distributed throughout the city except in its central and north-eastern areas. The exception is the group of the five census zones located adjacent to the northeast border of the study area. This group of census zones corresponds mostly to shanty towns still located adjacent to well-off areas. In turn, cluster 8, spread in various groups of census zones located mostly in the west and south part of the city, is the most critical cluster, socioeconomically vulnerable and with the highest percentage of diabetes.

## Discussion

SOM is classified as a soft clustering method, in contrast to k-means, which is a hard clustering method. Soft methods perform better than hard methods when dealing with fuzzy data, that is, the case when some points are difficult to classify in just one single cluster. In soft methods, these points may belong to more than one cluster with certain weights or probabilities. This fuzzy behaviour is frequent in large cities where high socioeconomic heterogeneity is often present. This characteristic of SOM turns out to be crucial for the analysis of our clusters. For example, clusters 3 and 8 are made up of people with similar socioeconomic attributes, but with different levels of diabetes (see Table in Appendix I). Perhaps, with a traditional hard algorithm, clusters 3 and 8 would probably merge into one single cluster or have different shapes and sizes. Put differently, SOM allows people who share some features but respond differently to the same input to split into two or more clusters. This fuzzy-based classification is illustrated by the colour gradient of the 2D grid representation in Fig. 1, as well as by the plane heat maps in Appendix II.

Exploring what makes a person more, less, or equally prone to develop a chronic disease goes beyond the scope of this study; however, we hope our results may help planners to deal with the optimisation of resources and allocation of health services. For example, people from cluster 8 are more prone to develop diabetes than people from cluster 3 despite their socioeconomic similarity. From these facts, one could hypothesise that such a difference is due to the lack of prevention campaigns, problems with the capacity or the location of health services, or some inherent attributes of people from such clusters not captured in the data, or even a mix of both aspects. In either case, further studies and/or new types of data are needed to find additional drivers or to investigate to what extent such drivers affect the prevalence of diabetes. Similarly, the main distinguishing feature of clusters 1 and 6 is accessibility to primary public health centres. Thus, one could be tempted to suggest the allocation of new health services in cluster 1. However, people from cluster 1, due to a privileged economic situation, are served in private and more expensive health services within the municipality they reside.

The methodology we suggest can be applied to investigate the relationship between chronic diseases and socioeconomic characteristics worldwide. However, it is based on a statistical method, and, as such, it may have some limitations that must be taken into account in the analysis. The first is the quality of the data. In our case, the CASEN survey is, in general terms, a trustworthy source of data with a robust sample design. However, the budget of socioeconomic surveys is often limited (at least in comparison with census surveys), so the small sample size may lead to biased results. If a bigger sample size is not possible, modellers should pay close attention to the sampling method used in the survey as well as the whole sample design process. Another point to bear in mind is the initial values of the SOM method. Random values are a good choice as a first attempt, but other choices can also be investigated in future studies. One option is the use of a method called *linear initialization* by which eigenvalues and eigenvectors are obtained from a principal component analysis. Kohonen [38] argues that the initial random vectors were originally used to demonstrate the capability of the SOM to become ordered, starting from an arbitrary initial state. Depending on the characteristics or the quality of the data, further studies could investigate whether random initial values or the linear initialisation ultimately converge to the same result. It may occur that some methods are more appropriate for some city or country, while not appropriate for others.

## Conclusion

In this study, we have combined two statistical techniques: spatial microsimulation and self-organising maps to explore the relationship between diabetes and socio-demographics in Santiago, Chile. The results have allowed us to corroborate the importance of the spatial factor in the analysis of chronic diseases as a way of suggesting differentiated solutions to spatially explicit problems; SOM turned out to be a good choice to deal with fuzzy health and socioeconomic data. The method explored and uncovered valuable spatial patterns for health decision-making. In turn, spatial microsimulation allowed the generation of the disaggregated data in a statistically meaningful manner.

We hope that the methodological aspects developed in this study at the neighbourhood level (small areas) can be complemented with other advanced methods, such as spatial econometrics, to investigate the causalities and drivers of chronic diseases. We also expect that our approach may help to shed some light on which group of people needs special attention, further analysis, and different solutions. Presenting our results in a spatially explicit manner and integrated into a geographical information system may help planners to find such solutions.

## Figures and Tables

**Fig. 6:**
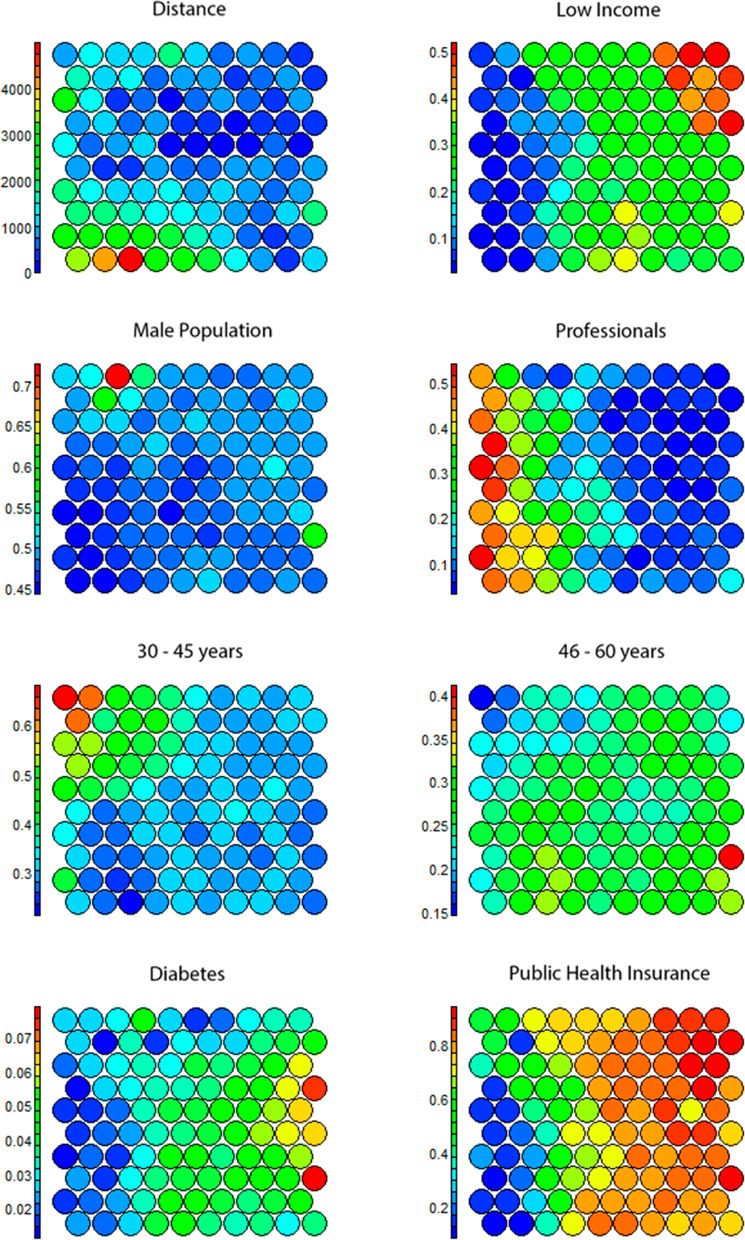
Plane heat maps of input attributes

## Data Availability

CASEN data can be obtained from the Ministry of Social Development and Family (http://observatorio.ministeriodesarrollosocial.gob.cl/) Census data can be obtained from the National Institute of Statistics (https://www.censo2017.cl/)

## Appendices

### Appendix I

See Table 2.

### Appendix II

See Fig. 6.

## References

[1] WHO. The world health report 2002 - Reducing risks, promoting healthy life. Education for Health. Geneve; 2002. 10.1080/1357628031000116808 14741909

[2] WHO. World health statistics 2018: monitoring health for the SDGs, sustainable development goals. Geneve; 2018.

[3] Nugent R (2008). Chronic diseases in developing countries: health and economic burdens. Ann N Y Acad Sci.

[4] Nelson K, Chapko M, Reiber G, Boyko E (2005). The association between health insurance coverage and diabetes care; data from the 2000 Behavioural Risk Factor Surveillance System. Health Serv Res.

[5] Rahman S, Mirza AS, Wathington D, Green S, Mayers Y, Iranmanesh E, et al. Chronic disease and socioeconomic factors among uninsured patients: a retrospective study. Chronic Illn. 2019. 10.1177/1742395319828430 30782011

[6] Minicuci N, Biritwum RB, Mensah G, Yawson AE, Naidoo N, Chatterji S, et al. Sociodemographic and socioeconomic patterns of chronic non-communicable disease among the older adult population in Ghana. Glob Health Action. 2014. 10.3402/gha.v7.21292 PMC3991840 24746141

[7] Pfeiffer DU, Robinson TP, Stevenson M, Stevens KB, Rogers DJ, Clements ACA (2008). Spatial analysis in epidemiology spatial analysis in epidemiology.

[8] Roquette R, Painho M, Nunes B (2017). Spatial epidemiology of cancer: A review of data sources, methods and risk factors. Geospat Health..

[9] Barnett JR (1993). Does the geographic distribution of physicians reflect market failure?: an examination of the New Zealand Experience, 1981–87. Environ Plan A Econ Sp..

[10] McIsaac M, Scott A, Kalb G (2015). The supply of general practitioners across local areas: accounting for spatial heterogeneity. BMC Health Serv Res..

[11] Roy S (1984). Demography of sterilization: Indian experience. Janasamkhya..

[12] Santow MG (1978). A microsimulation of Yoruba fertility. Math Biosci.

[13] Chernick H, Holmer M, Weinberg D (1987). Tax policy toward health insurance and the demand for medical services. J Health Econ..

[14] Parkin D (1985). A computer simulation model for the practical planning of cervical cancer screening programmes. Br J Cancer.

[15] Schneider U, Kleindienst J (2016). Monetising the provision of informal long-term care by elderly people: estimates for European out-of-home caregivers based on the well-being valuation method. Heal Soc Care Commun.

[16] Schofield D, Shrestha RN, Cunich MM, Passey ME, Veerman L, Tanton R (2017). The costs of diabetes among Australians aged 45–64-years from 2015 to 2030: Projections of lost productive life years (PLYs), lost personal income, lost taxation revenue, extra welfare payments and lost gross domestic product from Health&WealthMOD2030. BMJ Open..

[17] Singh P, Hussain R, Khan A, Irwin L, Foskey R. Dementia care: Intersecting informal family care and formal care systems. J Aging Res. 2014. 10.1155/2014/486521 PMC3950589 24701350

[18] Kohonen T (1982). Self-organized formation of topologically correct feature maps. Biol Cybern.

[19] Collan M, Eklund T, Back B (2007). Using the self-organizing map to visualize and explore socio-economic development. EBS Rev..

[20] Basara HG, Yuan M (2008). Community health assessment using self-organizing maps and geographic information systems. Int J Health Geogr..

[21] Mehmood Y, Abbas M, Chen X, Honkela T. Self-Organizing maps of nutrition, lifestyle and health situation in the world. In: Lecture Notes in Computer Science, vol 6731. Berlin: Springer; 2011.

[22] Wickramasinghe K, Alahakoon D, Schattner P, Georgeff M, Wang D, Reynolds M (2011). Self-organizing maps for translating health care knowledge: a case study in diabetes management. Lecture Notes in Computer Science (including subseries Lecture Notes in Computer Science, Advances in Artificial Intelligence.

[23] OECD. What´s happening with income inequality. In: Income Inequality: The Gap between Rich and Poor. Paris: OECD Publishing; 2015.

[24] OECD Group (2019). OECD reviews of public health: chile: a healthier tomorrow.

[25] Minsal. Encuesta Nacional de Salud 2016-2017 Segunda entrega de resultados. 2018. https://www.minsal.cl/wp-content/uploads/2018/01/2-Resultados-ENS_MINSAL_31_01_2018.pdf. Accessed 17 Feb 2020.

[26] Bambs C, Bravo-Sagua R, Margozzini P, Lavandero S. Science and health policies to tackle chronic diseases in Chile. Trends in Endocrinology and Metabolism. 2020. p. 67–70. 10.1016/j.tem.2019.11.010 31859214

[27] Social MD. Casen 2017, metodología de diseño muestral. Santiago de Chile; 2018. http://observatorio.ministeriodesarrollosocial.gob.cl/casen-multidimensional/casen/docs/Diseno_Muestral_Casen_2017_MDS.pdf.

[28] Valdivieso V, Montero J (2010). El plan AUGE: 2005 al 2009. Revista médica de Chile..

[29] Sapunar J (2016). Epidemiología de la Diabetes Mellitus en Chile. Rev Médica Clin Las Condes..

[30] Crespo R, Hernandez I. On the spatially explicit Gini coefficient: the case study of Chile—a high-income developing country. Lett Spat Resour Sci. 2020.

[31] Tanton R, Kimberley E, Tanton R, Edwards K (2013). Spatial Microsimulation: a reference guide for users. Spatial microsimulation: a reference guide for Users.

[32] Zaidi A, Harding A, Williamson P, editors. New frontiers in microsimulation modelling: introduction. 1st ed. New Frontiers in microsimulation modelling. Routledge; 2009.

[33] Lovelance R, Dumont M. Spatial Microsimulation with R. Chapman and Hall/CRC; 2017. 260 p.

[34] Carrasco M, Brunner R (2013). SOMz: photometric redshift PDFs with self organizing maps and random atlas. Mon Not R Astron Soc..

[35] Voas D, Williamson P (2001). Evaluating goodness-of-fit measures for synthetic microdata. Geogr Environ Model..

[36] Smith D, Clarke G, Harland K (2009). Improving the synthetic data generation process in spatial microsimulation models. Environ Plan A..

[37] Wehrens R, Buydens L (2007). Self- and Super-Organizing Maps in R: the kohonen Package. J Stat Softw.

[38] Kohonen T (2013). Essentials of the self-organizing map. Neural Networks..

[39] Skific N, Francis J. Self-Organizing Maps: A Powerful Tool for the Atmospheric Sciences. In: Applications of Self-Organizing Maps. 2012.

[40] Theodoridis S, Koutroumas K (2003). Pattern Recognition.

[41] Nielsen F (2016). Introduction to HPC with MPI for Data Science.

